# ER‐α36 is involved in calycosin inhibition of IL‐6 production in macrophages

**DOI:** 10.1111/jcmm.18037

**Published:** 2023-11-16

**Authors:** Guoli Wu, Guangying Qi, Yu Liu, Jinfeng Gan, Chichu Xie, Qi Wu, Wei Cui, Chunhua Wang, Zhaoyi Wang

**Affiliations:** ^1^ Xiangya Hospital Central South University Changsha China; ^2^ Guangxi Key Laboratory of Tumor Immunology and Microenvironment Regulation, Department of Basic Medicine Guilin Medical University Guilin China

**Keywords:** calycosin, ER‐α36, IL‐6, macrophage, NF‐κB

## Abstract

The tumour microenvironment (TME) is crucial for tumour development and progression. Tumour‐associated macrophages (TAMs) in the TME can promote tumour progression and metastasis by releasing cytokines, such as IL‐6. Calycosin, a phytoestrogen that is one of the active compounds in *Radix Astragali*, has been shown to inhibit tumour growth and metastasis. However, the underlying mechanism by which calycosin inhibits tumour growth remains unclear. Thus, this study aimed to investigate the effect of calycosin on IL‐6 production in peripheral blood mononuclear cell (PBMC)‐ and THP‐1‐derived macrophages and explore its potential mechanisms using co‐immunoprecipitation, western blotting, immunofluorescence, chromatin immunoprecipitation and luciferase assays. We found that calycosin treatment substantially upregulated the expression of ER‐α36, a variant of the ER, and reduced IL‐6 production in macrophages. Mechanistically, ER‐α36 physically interacted with NF‐κBp65 and retained p65 in the cytoplasm to attenuate NF‐κB function as an IL‐6 transcriptional inducer. In conclusion, our result indicated that calycosin inhibited IL‐6 production by enhancing ER‐α36 expression and its interaction with p65, which attenuated NF‐κB function as an IL‐6 inducer. Therefore, calycosin can be developed as an effective agent for cancer therapy by targeting TAMs.

## 1 INTRODUCTION

Tumour microenvironment (TME) is the environment in which tumours develop, grow and metastasize.^1^ Thus, the TME is a key element in cancer progression, including tumour initiation, metastasis and resistance to treatment.^2^ The TME comprises cancer cells and various stromal cells, such as fibroblasts, neutrophils, macrophages and other immune cells.^3^ Tumour‐associated macrophages (TAMs) are an extremely heterogeneous population that display proinflammatory and anti‐inflammatory functions, accounting for 6%–14% of the total cells in the tumour tissue.^4^ Of the immune cells, TAMs are the most abundant and produce a variety of proinflammatory cytokines in the TME, such as IL‐6 and TNF‐α, and promote tumour cell growth.^5^ Along with other stromal cells, TAMs form an inflammatory microenvironment around the tumour.^6^ Therefore, therapeutic strategies targeting macrophages are appealing.

In the typical NF‐κB signalling pathway, NF‐κB is bound to an inhibitor known as IκB in an inactive form.^7^ IκB then transports activated NF‐κB from the cytoplasm to the nucleus, followed by releasing NF‐κB into the nucleus through the phosphorylation and degradation of IκB to activate NF‐κB transcriptional activity and inflammatory responses.^8^ The transcriptional activity of NF‐κB is a key factor in regulating the expression of numerous cytokine genes, such as *IL‐6*. As a well‐known pleiotropic cytokine,^9^ IL‐6 expression is induced in response to different extracellular cues, such as the activation of Toll‐like receptor 4 (TLR4) by lipopolysaccharide (LPS).^10^ One of the key proteins controlling IL‐6 expression is the transcription factor NF‐κB (a dimer composed of p65 (*RelA*) and p50 subunits).^11^

Through its receptors, oestrogen influences pathological and physiological processes in various cells and tissues.^12^ ER‐α is the major oestrogen receptor that is the product of ESR1. The *ESR1* gene not only serves as a template for a full‐length 66 kDa protein (ER‐α66) but also for two alternative isoforms (ER‐α46 and ER‐α36).^13^ In 2005, Dr. Wang's laboratory identified and cloned a novel subtype of ER‐α, ER‐α36. Different from the classic ER‐α66, ER‐α36 lacks both AF‐1 and AF‐2 domains of ER‐α66 and possesses an altered ligand‐binding domain.^13^ ER‐α36 is mainly located in the plasma membrane, mediating the nongenomic oestrogen signalling.^14^ Previous research has shown that a high level of ER‐α36 expression is associated with an aggressive phenotype in a variety of cancers, including breast cancer,^15^, ^16^ lung adenocarcinoma,^17^ endometrial cancer^18^ and gastric cancer.^19^ Previous reports have shown that ER‐α36 can inhibit LPS‐induced IL‐6 production from monocytes.^20^

Calycosin, a major isoflavonoid in *Radix Astragali*, exhibits potent anti‐inflammatory and antioxidant properties.^21^ In addition, calycosin exhibits various anticancer properties^22^, ^23^, ^24^; its mechanism of action, however, is not well understood.

Here, we used PBMC‐ and THP‐1‐derived macrophages in vitro and tumour‐bearing mice in vivo to investigate the function and underlying mechanism of calycosin‐mediated inhibition of IL‐6 production in macrophages as well as the possible involvement of ER‐α36.

## 2 MATERIALS AND METHODS

### 2.1 Reagents

The reagents used in the study were LPS and Phorbol 12‐myristate 13‐acetate (PMA; MedChemExpress); calycosin (≥98%, Sigma); DMEM and RPMI 1640 (Procell); antibodies against Rpb1, IKK, p65, IκB, p‐IKK, p‐p65 and p‐IκB (CST); antibodies against β‐actin and PARP1 (Proteintech); antibodies against ER‐α36 were generated by ABclonal using the last 20 amino acids of ER‐α36 as antigen; and Mouse F4/80 Positive Selection Kit and Human Monocyte Isolation Kit (STEMCELL Technologies).

### 2.2 Cell culture and calycosin treatment

Murine hepatocellular carcinoma Hepa1‐6 cells, human acute monocytic leukaemia THP‐1 cells and human hepatocellular carcinoma PLC/PRF/5 and BEL‐7402 cells were purchased from the Cell Resource Centre. BEL‐7402 and THP‐1 were cultured in RPMI‐1640 medium, whereas PLC/PRF/5 and Hepa1‐6 cells were cultured in DMEM. All the media were supplemented with 10% FBS (Thermo Fisher Scientific) and 100 μg/mL penicillin–streptomycin solution (Solarbio). THP‐1 cells were cultured in media containing 0.05 mM β‐mercaptoethanol (AbMole).

### 2.3 PBMC isolation and calycosin treatment

This study received approval from the Ethics Committee of Affiliated Hospital of Guilin Medical University. Peripheral venous blood was obtained from median cubital vein of healthy donors. The informed consent was obtained from six donors (four males and two females, mean age = 29.5 years). PBMCs were isolated from peripheral venous blood using monocyte isolation kit (STEMCELL Technologies). After centrifugation over a density gradient solution (STEMCELL Technologies), the PBMCs were washed twice with EasySep Buffer (STEMCELL Technologies). The PBMCs were enriched through negative selection using magnetic beads conjugated with a mixture of antibodies against CD4, CD8, CD19, CD56 and others.

The PBMCs were treated with 100 ng/mL macrophage colony‐stimulating factor (M‐CSF; PeproTech) for 4 days to induce macrophage differentiation. THP‐1 cells were treated with PMA (100 ng/mL) for 48 h to induce macrophage differentiation. The induced macrophages were treated with calycosin (10 nM) for 12 h and activated with 1 μg/mL LPS for another 12 h. The supernatants were collected as calycosin‐treated conditioned medium, and the IL‐6 level in the individual supernatant samples was assessed.

### 2.4 RNA isolation and RT‐PCR

Total RNA was extracted from the different groups of cells or tissue homogenates using TRIzol reagent (TIANGEN), and after qualification and quantification of RNA samples using the NanoDrop one spectrophotometer (Thermo Fisher Scientific), the RNA samples were reverse‐transcribed into cDNA using a cDNA synthesis kit (Sangon Biotech). The relative level of the gene of interest to the control β‐actin mRNA transcripts in individual samples was quantified via RT‐PCR in a CFX96 PCR machine (Bio‐Rad) using regular reagents, including specific primers (Appendix S1). The data were analysed using the 2^−ΔΔCt^ method.

### 2.5 Co‐immunoprecipitation (Co‐IP)

The cells were collected and centrifuged at 1000 × *g* for 3 min, the supernatant was discarded, and the prechilled weak lysate buffer (Solarbio) was added, mixed and centrifuged at 12,000 × *g* for 10 min. The supernatants were then transferred to new tubes. Approximately 5% of the supernatant of cell lysis was boiled in 4× loading buffer for 5 min and used as the input, while the rest of the supernatant was incubated with anti‐ER‐α36 or p65 antibody or IgG at 4°C for 4 h, and centrifuged at 1400 × *g* for 3 min. Protein A/G agarose beads (Santa Cruz Biotechnology) were added to the samples to pull down the immunocomplexes. Finally, the supernatant was discarded and the protein precipitates were boiled in 4× loading buffer for 5 min to prepare the Co‐IP samples.

### 2.6 Lentivirus infection and RNA interference

Full‐length ER‐α36 cDNA was cloned into the pCDH‐CMV lentivirus plasmid (Addgene), and the construct was validated via sequencing. To generate the recombinant lentivirus, the construct was co‐transfected with lentiviral packaging vectors (pMD2.G and psPAX2) into HEK‐293 T cells using Lipofectamine 3000 (Invitrogen). After 2 days, the supernatant was collected to transduce THP‐1 cells. For transduction of THP‐1 cells, the lentiviral supernatant was added into the THP‐1 culture medium for 48 h. Puromycin (5 μg/mL; Solarbio) was then used to select the infected THP‐1 cells. The lentiviral infection efficiency was monitored using GFP fluorescence. The surviving clones were pooled and detected using western blotting to ensure ER‐α36 overexpression.

An siRNA was used to downregulate ER‐α36 expression. THP‐1 cells were transfected with control siRNA or siRNA‐ER‐α36 (50 nM) using Lipofectamine 3000 (Invitrogen). The transfection medium was replaced with fresh medium after 6 h. At 48‐h post‐transfection, the efficiency of siRNA interference was evaluated via western blotting. The target sequences used for siRNA against ER‐α36 were synthesized by General Biol as shown in the Appendix S1.

### 2.7 Luciferase assay

THP‐1 cells were transfected with the plasmids pRL‐TK Renilla (Beyotime) and pNF‐κB‐TA‐luc (Beyotime) using Lipofectamine 3000 (Invitrogen). Forty‐eight hours later, lysis buffer was used to lyse the cells, which were then centrifuged at 12,000 × *g* for 5 min. To collect the supernatant, the cell lysates were first mixed with 100 μL of firefly luciferase detection reagent (Beyotime) and measured for relative light units (RLUs), and then with 100 μL of Renilla luciferase assay solution, and the RLU was measured again. The ratio of the RLU of firefly luciferase to the RLU of Renilla luciferase is shown as relative luciferase activity.

### 2.8 Tumour assay in vivo

This study was approved by the Animal Ethics Committee of the Guilin Medical University. All experiments were performed in accordance with the National Institutes of Health Guide for the Care and Use of Laboratory Animals. Male BALB/c mice (28–35 D old, weighing 14–18 g, *n* = 16) were obtained from Hunan Slake Jingda Experimental Animal Co., Ltd. All mice were housed in a specific pathogen‐free laboratory at 20–25°C and a cycle of 12‐h dark/light, suitable humidity and free access to normal mouse chow and water. Individual mice were inoculated subcutaneously with ~5 × 10^6^ Hepa1‐6 cells in their left armpits. One day later, the mice were randomized and injected intraperitoneally with PBS as the control or 50 μg/kg body weight of calycosin daily for 30 consecutive days as the calycosin group (*n* = 8 per group). At the end of the experiment, venous blood samples were collected, anaesthetized using sodium pentobarbital and euthanized via cervical dislocation. The tumours were dissected, imaged and measured. The level of IL‐6 expression in the tumour tissues and blood samples was quantified using ELISA and RT‐PCR. Macrophage infiltrates in the tumour tissues were also isolated and examined.

### 2.9 Mouse tumour‐infiltrating macrophage isolation

Macrophage infiltrates in the tumour tissues were isolated using a Mouse F4/80 Positive Selection Kit (STEMCELL Technologies) according to the manufacturer's instructions. Briefly, tumour tissues were harvested, minced and digested with a digestion medium containing collagenase/hyaluronidase, DNase I and RPMI 1640 medium, followed by filtering through a nylon mesh strainer. After washing, the cells were suspended and cultured in 20 mL of ammonium chloride for 5 min. Macrophages were purified using magnetic particles conjugated to the F4/80 antibody. The interaction between p65 and ER‐α36 in the macrophages from tumour tissues was analysed using co‐IP, and the level of NF‐κB signalling even expression in macrophages was examined via western blotting. p65 level in the cytoplasm and nucleus of macrophages was analysed.

### 2.10 Statistical analysis

Data are expressed as the mean ± the standard error of the mean (SEM) of each group from at least three separate experiments. Data were analysed using Student's *t*‐test or anova using SPSS software. The mean and standard deviation are shown as means ± SEM. Statistical significance was set at *p* < 0.05 (Additional information is shown in the Supplemental Materials and Methods in Appendix S1).

## 3 RESULTS

### 3.1 TAM infiltration analysis

**FIGURE 1:**
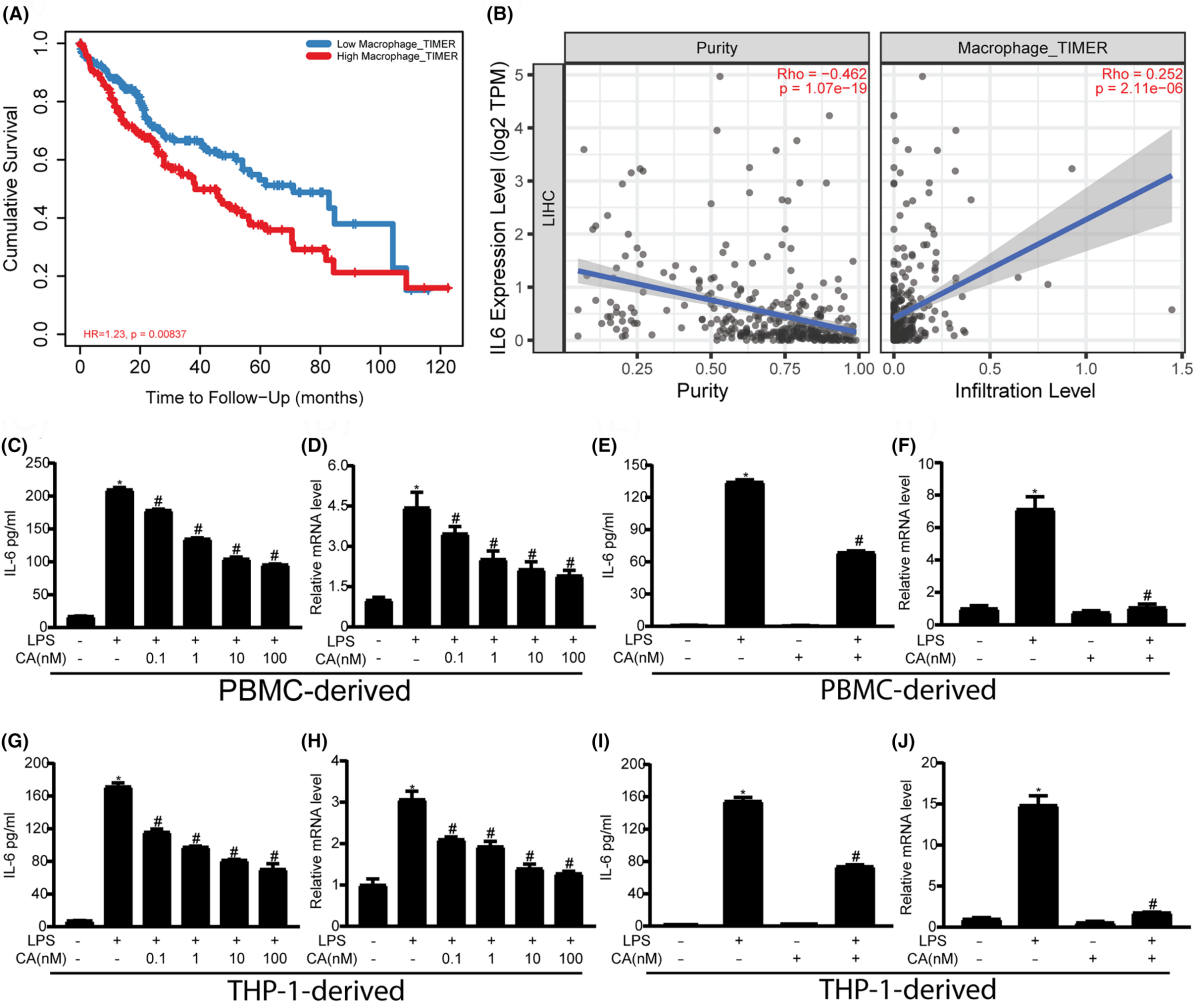
Calycosin inhibits LPS‐induced production of IL‐6 in macrophages. (A). A survival curve of the patients carrying liver hepatocarcinoma (HCC) infiltrated with high or low TAMs. (B). Correlation between IL‐6 and liver HCC cells in the patients (left). Correlation between IL‐6 and TAMs in the patient with liver HCC (right). ELISA and RT‐PCR analysis of IL‐6 expression in PBMC‐ and THP‐1‐derived macrophages treated with LPS in the presence or absence of calycosin. PBMC‐derived macrophages (C–F) and THP‐1‐derived macrophages (G–J). IL‐6 expression in PBMC‐derived macrophages (C and E) and THP‐1‐derived macrophages (G and I) were detected using ELISA. RT‐PCR analysis of IL‐6 expression in PBMC‐derived macrophages (D and F) and THP‐1‐derived macrophages (H and J). *p < 0.05 vs. the control group (LPS‐ and calycosin‐), ^#^ *p* < 0.05 vs. the LPS group (LPS 1 μg/mL and calycosin‐).

A previous study has shown that IL‐6 secreted by TAMs promotes tumour growth.^25^ We investigated the effects of macrophage infiltration and IL‐6 expression on the development of human hepatocellular carcinoma (HCC). TIMER algorithm was used to study the potential relationship between macrophage infiltration and HCC development. Analysis of the data from TIMER 2.0 showed that high macrophage infiltration resulted in poorer survival than low macrophage infiltration (Figure 1A). In addition, a significant positive correlation was noted between IL‐6 expression and the extent of tumour‐infiltrating macrophages in liver HCC (Figure 1B), suggesting that macrophage infiltration and IL‐6 expression promote the progression of human HCC.

### 3.2 Calycosin inhibited LPS‐induced IL‐6 production

Calycosin inhibits HCC growth.^26^ Antitumour agents, including those involved in cell growth, apoptosis and metastasis, have been studied directly in tumour cells. However, only few studies have investigated the effects of these agents on TAMs. Because macrophage‐secreted IL‐6 is involved in tumour progression, we decided to determine the effect and underlying mechanism of calycosin action on IL‐6 production in macrophages. LPS readily induced IL‐6 production in PBMC‐derived macrophages and macrophages derived from THP‐1 cells, as revealed using ELISA (Figure 1C,G), in a concentration‐dependent manner (Figure 1E,I). Therefore, 10 nM calycosin was selected for further studies. We further found that the mRNA level of IL‐6 was also reduced by calycosin, as found using RT‐PCR (Figure 1D,F,H,J), suggesting that the effect of calycosin on IL‐6 expression occurred at the transcriptional level. The growth of these macrophages was not significantly affected by 10 nM calycosin, as determined using the MTT assay (Figure S1), suggesting that growth inhibition was not involved. Thus, our results indicate that calycosin inhibited LPS‐induced IL‐6 expression in macrophages.

### 3.3 Calycosin suppressed the growth‐promoting activity of IL‐6 from macrophages in HCC cells

**FIGURE 2:**
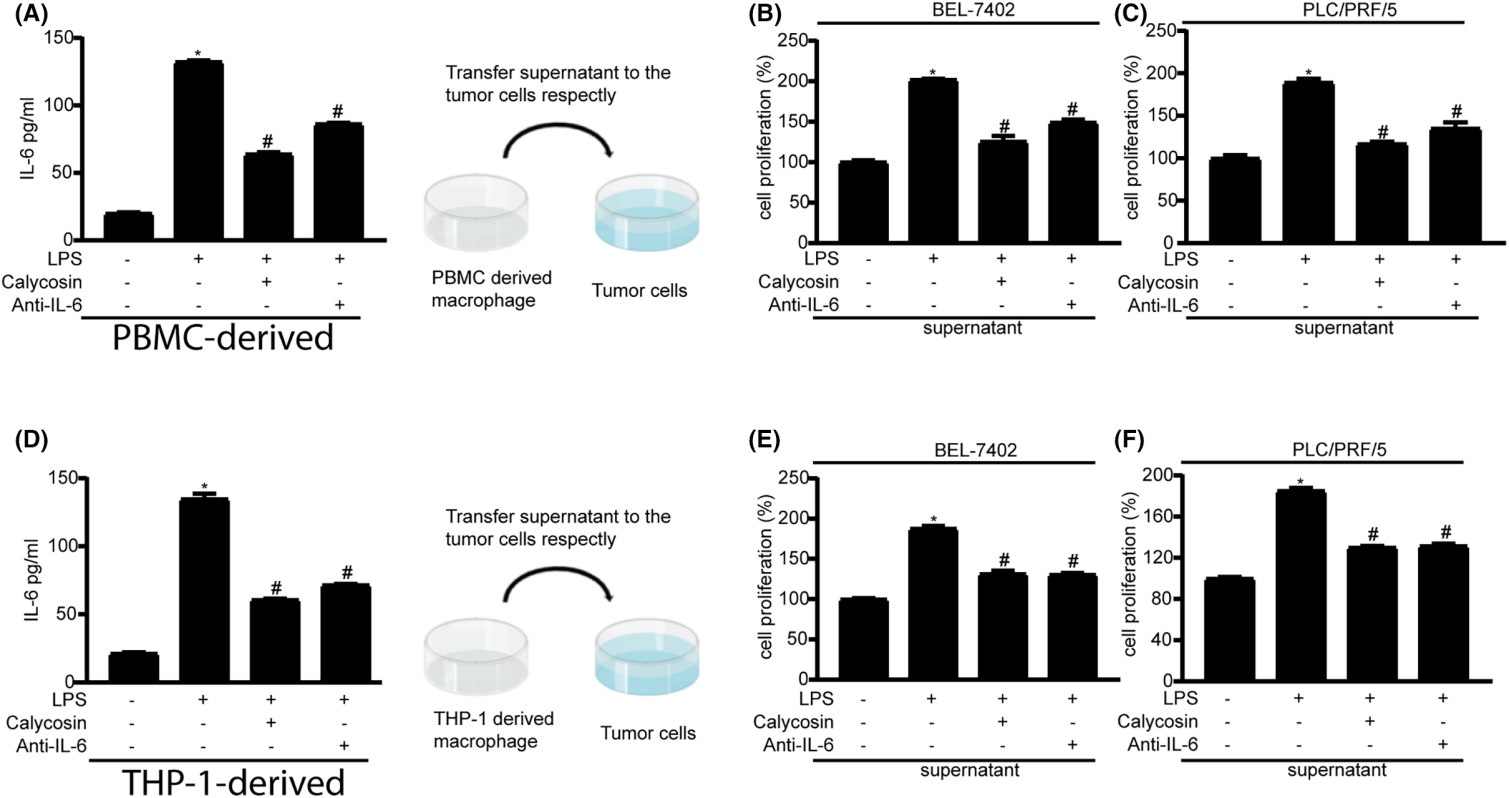
Supernatants from PBMC‐ and THP‐1‐derived macrophages stimulate HCC cell growth (A and D). ELISA of IL‐6 expression in PBMC‐ and THP‐1‐derived macrophages treated with LPS or calycosin in the presence or absence of the IL‐6 neutralizing antibody (B and C; E and F). MTT assay of the HCC cells treated with the supernatant from the PBMC‐ and THP‐1‐derived macrophages in the presence and absence, respectively, of anti‐IL‐6 neutralizing antibody. Data are shown as the mean ± SEM of three independent experiments, *p < 0.05 vs. the control group (LPS‐, calycosin‐), ^#^ *p* < 0.05 vs. the LPS group (LPS 1 μg/mL and calycosin‐).

IL‐6 also promotes tumour progression.^27^ We investigated the effects of calycosin on IL‐6 growth‐promoting activity and found that IL‐6 level in the supernatant of LPS‐treated macrophages increased, as revealed using ELISA (Figure 2A,D); however, IL‐6 function was blocked by an anti‐IL‐6 blocking/neutralizing antibody, as shown in Figure 2A,D. After PLC/PRF/5 and BEL‐7402 cells were incubated with different supernatants from macrophages for 48 h, the viability of BEL‐7402 and PLC/PRF/5 cells was examined using MTT assay (Figure 2B,C,E,F). The supernatant from LPS‐treated cells stimulated the growth of both cell lines, whereas the supernatant from LPS‐treated cells pretreated with IL‐6 blocking or neutralizing antibody failed to do so. In addition, the supernatant from the calycosin‐treated macrophages failed to stimulate HCC cell growth. These results indicate that IL‐6 secreted from LPS‐treated macrophages stimulated HCC cell growth, which was attenuated by calycosin treatment.

### 3.4 NK‐κB signalling pathway and ER‐α36 in calycosin inhibition of IL‐6 production from LPS‐induced macrophages

**FIGURE 3:**
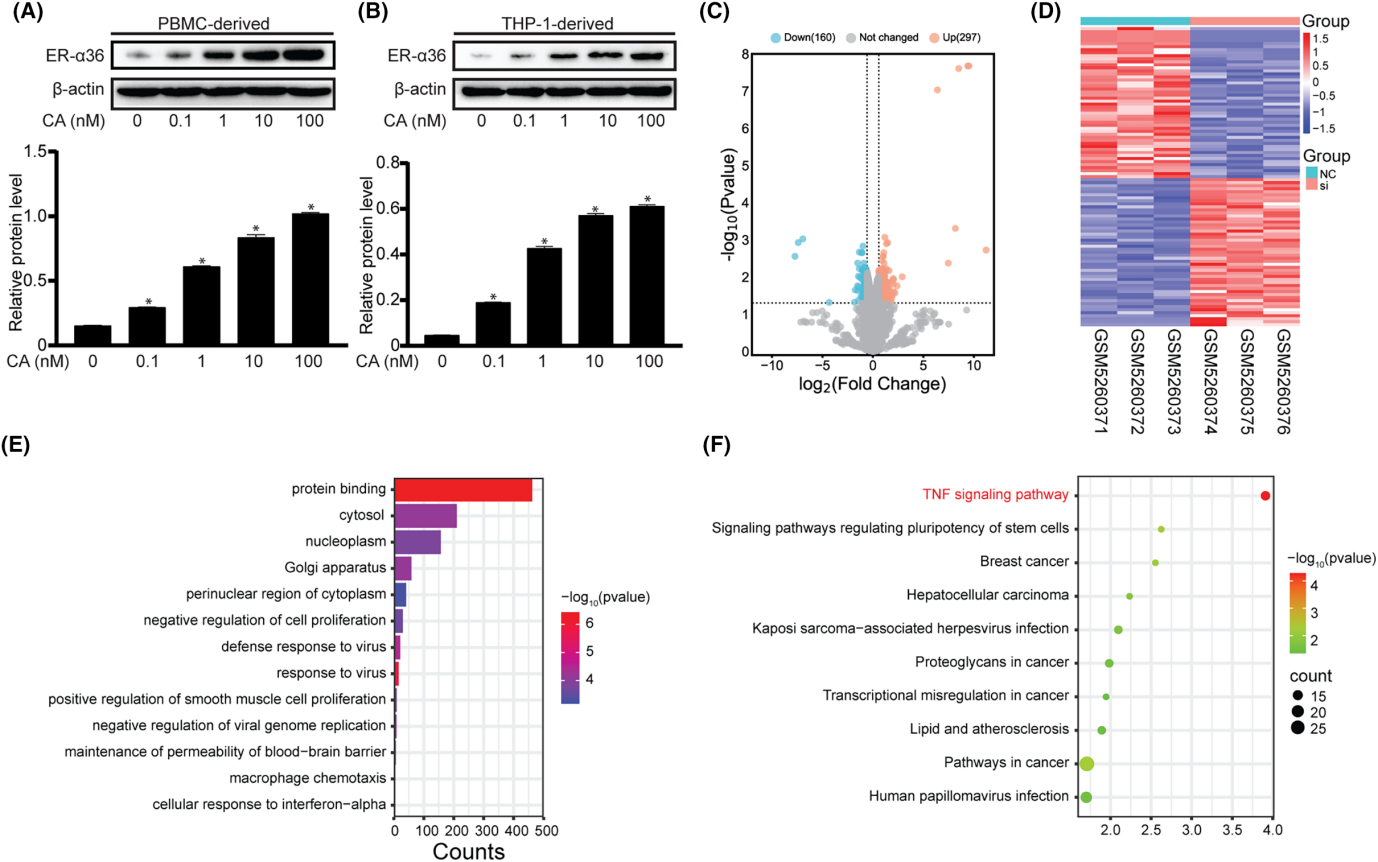
Calycosin induces ER‐α36 expression in macrophages. (A and B) ER‐α36 expression in THP‐1‐ and PBMC‐derived macrophages treated with or without calycosin was assessed via western blotting. Protein bands were semiquantified via densitometric analysis. Data are shown as the mean ± SEM of three independent experiments, *p < 0.05 vs. the control group (0 nM). (C) Differentially expressed genes from the CasKi cells transfected with si‐NC and si‐ERα‐36 siRNA are shown using a gene volcano map. (D) The top 50 genes with the most significant upregulation and downregulation are presented in the heatmap. (E and F). The total number of DEGs was used to perform GO and KEGG enrichment.

Previous studies have suggested that ER‐α36 is associated with IL‐6 secretion in monocytes treated with oestrogens.^20^ As calycosin has been known as a phytoestrogen for a long time, we decided to examine the effect of calycosin on ER‐α36 expression in PBMC‐ and THP‐1‐derived macrophages. We found that calycosin potently upregulated ER‐α36 expression in a dose‐dependent manner in the presence or absence of LPS (Figure 3A,B and Figure S2).

Molecular docking was used to mimic the interaction between ER‐α36 and molecules, including oestrogen, formononetin, biochanin A and calycosin. We found that all molecules bound to ER‐α36, among which calycosin had the second strongest binding affinity after oestrogen (Figure S3), suggesting that calycosin upregulates ER‐α36, presumably by binding to ER‐α36.

To probe the signalling pathway downstream of ER‐α36 that is involved in calycosin activity in macrophages, we analysed data from ER‐α36 knock‐down cell lines in the GEO database. The DEGs are displayed as volcano plots in Figure 3C. The top 50 genes with the most significant upregulation and downregulation are shown in a heatmap (Figure 3D). GO annotation and enrichment analyses of DEGs were performed (Figure 3E). KEGG enrichment analysis was also conducted for 457 DEGs, and the results revealed 10 pathways enriched for differentially expressed proteins (Figure 3F). These pathways mainly concern ‘TNF signalling pathway’.

**FIGURE 4:**
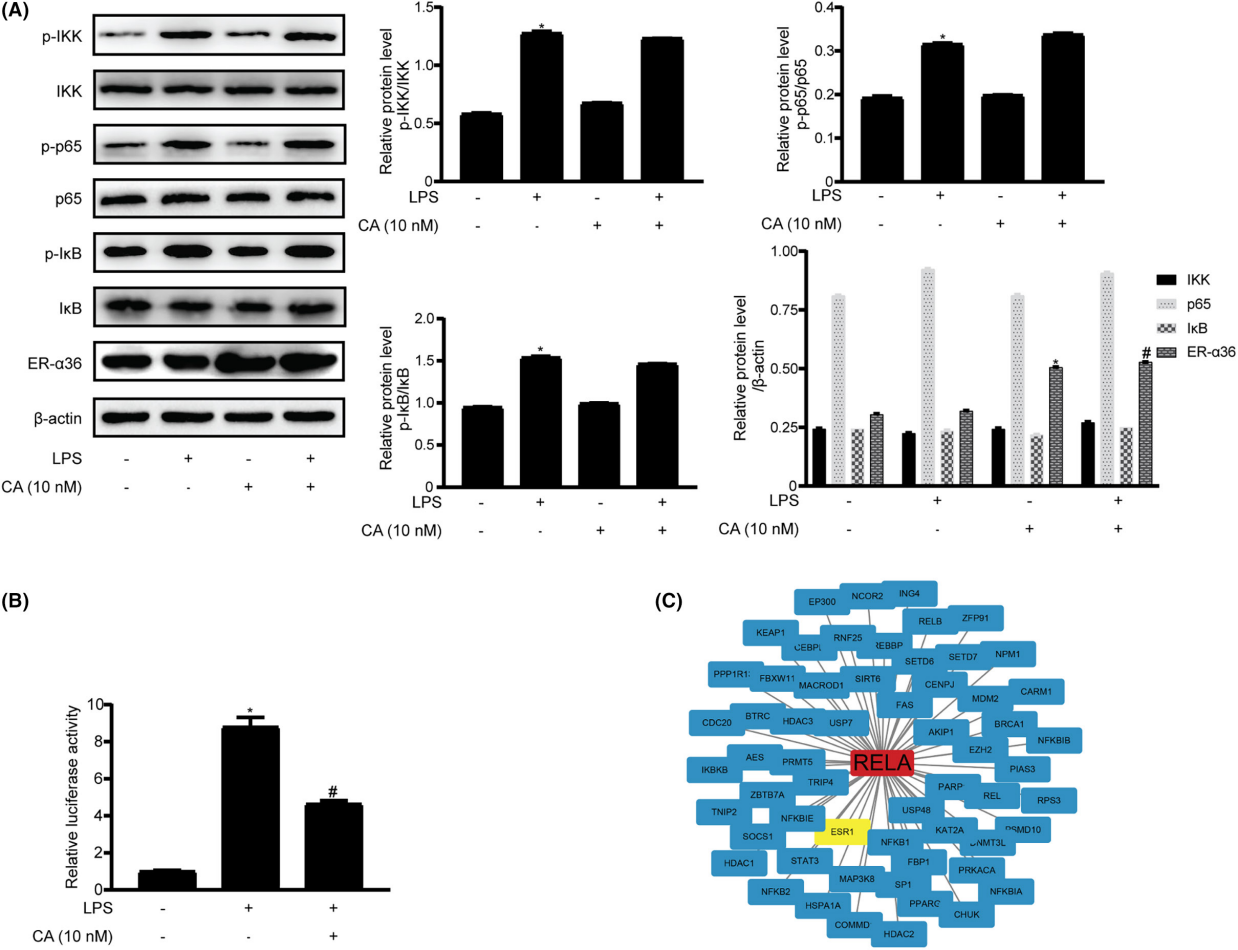
Calycosin induces NF‐κB transcription activity in macrophages. (A) The expression level of the proteins and their phosphorylated forms of the NF‐κB signalling pathway in THP‐1 cells were assayed via western blotting. Protein bands were semiquantified via densitometric analysis. Data are shown as the mean ± SEM of three independent experiments, *p < 0.05 vs. the control group (LPS‐ and calycosin‐), ^#^ *p* < 0.05 vs. the LPS group (LPS 1 μg/mL and calycosin‐). (B) Dual‐luciferase assay of NF‐κB transcriptional activity of THP‐1. All experiments were repeated thrice, *p < 0.05 vs. the control group (LPS‐ and calycosin‐), ^#^ *p* < 0.05 vs. the LPS group (LPS 1 μg/mL and calycosin‐). (C) BioGrid software predicted that p65 (*RELA*) may interact with oestrogen receptor.

As the NF‐κB signalling pathway is one of the important downstream cascades of ‘TNF signalling pathway’, we decided to explore whether the NF‐κB pathway is involved in calycosin inhibition of IL‐6 production in LPS‐treated macrophages. The expression level of IKK, p65 and IκB proteins as well as their phosphorylated forms was examined via western blotting. Compared with those in the control group, the expression level of IKK, p65 and IκB proteins was without change in the LPS group, whereas the level of phosphorylated IKK, p65 and IκB proteins was increased (Figure 4A). In the calycosin group, no changes were observed in the expression level of IKK, p65 and IκB, whereas phosphorylated IKK, p65 and IκB proteins appeared less evident than those in the LPS group.

As LPS induces IL‐6 production in an NF‐κB‐dependent manner in macrophages, we examined whether LPS is also involved in calycosin activity in macrophages. We co‐transfected THP‐1 cells with pNF‐κB‐TA‐luc and pRL‐TK Renilla as a transfection efficiency control. We found that LPS stimulation augmented NF‐κB‐driven transcriptional activity, which was attenuated by calycosin (Figure 4B), indicating that calycosin inhibits IL‐6 production by downregulating NF‐κB transcription signalling.

### 3.5 ER‐α36 interacted with p65, retained p65 in the cytoplasm and inhibited IL‐6 production in LPS‐induced macrophages

**FIGURE 5:**
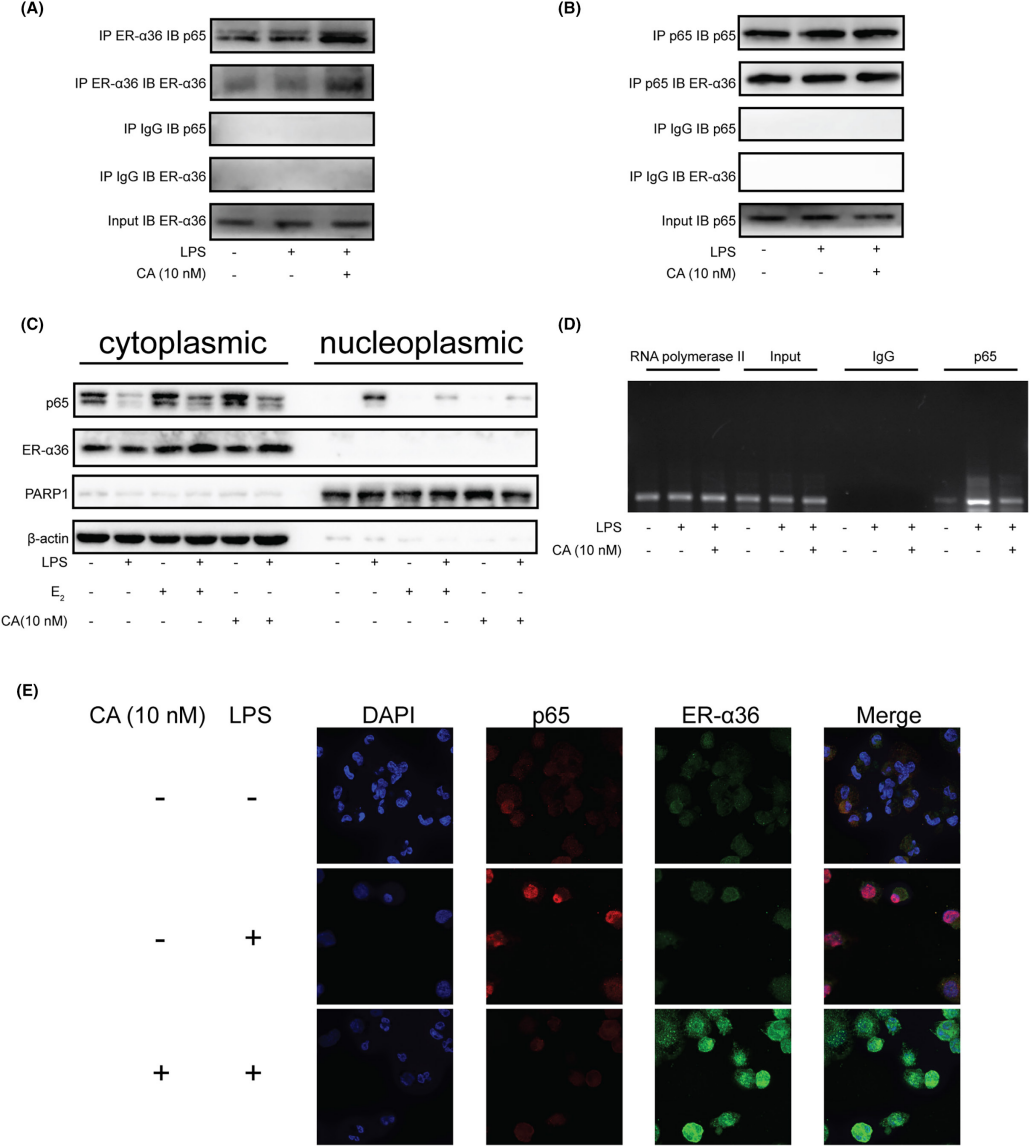
ER‐α36 interacts with p65, retains p65 in the cytoplasm and inhibits IL‐6 production induced by LPS in macrophages. (A and B) Co‐immunoprecipitation confirmed the interaction between ER‐α36 and p65. Lysates of THP‐1‐derived macrophage treated with LPS in the presence or absence of calycosin were immunoprecipitated either with an ER‐α36 antibody or a p65 antibody. A negative control was performed using IgG. Pulled‐down proteins were probed for the presence of p65 or ER‐α36 via western blotting. (C) The nucleoplasmic and cytoplasmic separation assay of ER‐α36 and p65 proteins in LPS‐treated macrophages, oestrogen (E_2_), and calycosin (CA). PARP1 and β‐actin antibodies were used as nucleoplasmic and cytoplasmic markers, respectively. (D) Calycosin inhibited p65 binding to the IL‐6 promoter. Cross‐linked chromatin was immunoprecipitated with rabbit IgG, anti‐Rpb1 and anti‐p65 antibodies. Eluates of the immunoprecipitated DNA samples were PCR‐amplified with a primer pair specific to the IL‐6 promoter region. (E) Immunofluorescence assay of ER‐α36 (green) and p65 (red) in the macrophages treated with LPS and calycosin (CA).

Biogrid analysis suggested that p65 (*RelA*) interacts with ESR1 (Figure 4C). Therefore, we examined the interaction between p65 and ER‐α36 in macrophages treated with calycosin. Immunoprecipitation of ER‐α36 from the cell extracts treated with calycosin efficiently reduced p65 (Figure 5A). Similarly, p65 immunoprecipitation also co‐immunoprecipitated ER‐α36 (Figure 5B), indicating that ER‐α36 physically interacted with the NF‐κB subunit p65.

In addition, we reasoned that calycosin induces ER‐α36 protein expression and promotes its interaction with p65, which may retain p65 in the cytoplasm of macrophages. As shown in Figure 5C, LPS stimulation facilitated p65 nuclear translocation in macrophages, whereas calycosin treatment substantially decreased the nuclear localization of p65. Similar results were obtained for oestrogen, suggesting that the phytoestrogen hindered p65 nuclear translocation.

Immunofluorescence assay was performed to confirm these results. As shown in Figure 5E, p65 (red) was mainly localized in the nucleus of macrophages after LPS treatment and was blocked by calycosin treatment. In addition, ER‐α36 expression (green) was enhanced and co‐localized with p65 in the cytoplasm of the macrophages treated with LPS and calycosin. These results further indicate that calycosin enhances ER‐α36 expression and retains p65 in the cytoplasm of calycosin‐treated macrophages.

Finally, we used chromatin immunoprecipitation to directly assess the effect of calycosin on the binding of p65 to the promoter region of *IL‐6* gene. We found that calycosin treatment attenuated p65 binding to the *IL‐6* promoter (Figure 5D), suggesting a mechanism by which calycosin inhibits IL‐6 secretion from LPS‐treated macrophages.

### 3.6 ER‐α36 in calycosin inhibited IL‐6 production in LPS‐treated macrophages

**FIGURE 6:**
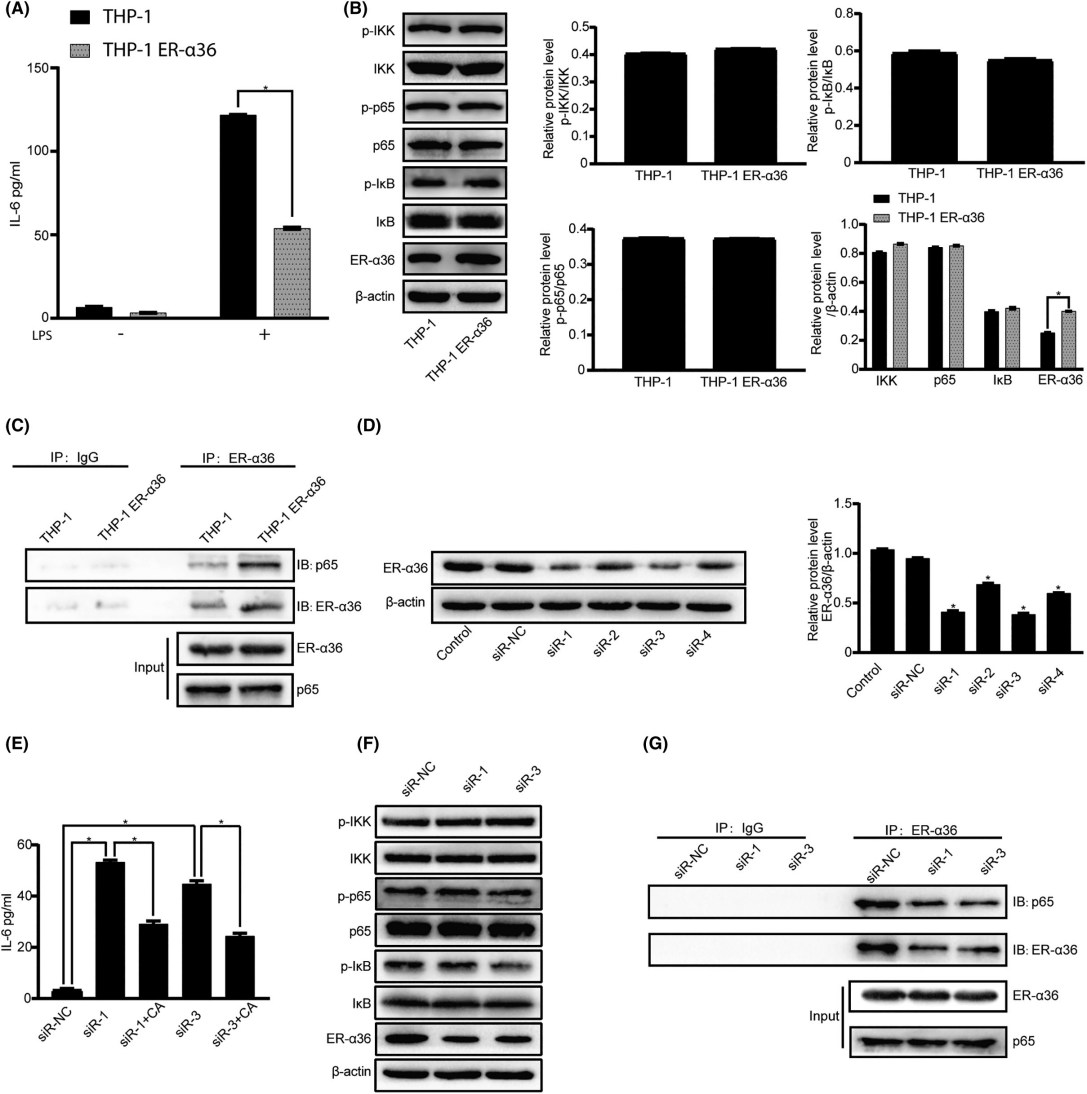
ER‐α36 is involved in calycosin inhibition of IL‐6 production from the macrophages treated with LPS. (A) IL‐6 production in the THP‐1‐derived macrophages with or without ER‐α36 overexpression was analysed using ELISA. Data are shown as the mean ± SEM of three independent experiments. *p < 0.05 vs. the cells with ER‐α36 overexpression (LPS 1 μg/mL). (B) Western blotting of the expression level of the proteins and their phosphorylated forms in the NF‐κB signalling pathway and the level of ER‐α36. Protein bands were semiquantified via densitometric analysis. *p < 0.05. (C) Co‐immunoprecipitation of the lysates from the macrophages with or without forced ER‐α36 expression using an anti‐ER‐α36 antibody for IP and anti‐p65 antibody for western blotting. (D) ER‐α36 protein expression in THP‐1‐derived macrophages transfected with siRNA, as determined via western blotting. * p < 0.05 vs. the control group. (E) ELISA of IL‐6 expression in the macrophages transfected with siRNA against ER‐α36 (siR‐1 and siR‐3) in the presence or absence of calycosin (10 nM). Data are shown as the mean ± SEM of three independent experiments. *p < 0.05. (F) Western blotting of the expression level of NF‐κB signalling proteins in THP‐1‐derived macrophages after ER‐α36 knock‐down. (G) Co‐immunoprecipitation of the lysates from the macrophages with or without ER‐α36 knock‐down using an anti‐ER‐α36 antibody for IP and an anti‐p65 antibody for western blotting.

To directly examine the involvement of ER‐α36 in calycosin activity in macrophages, we forced ER‐α36 overexpression in human THP‐1 cells through infection with the lentivirus containing the full‐length ER‐α36 cDNA. We found that ER‐α36 overexpression resulted in a substantial reduction in IL‐6 production in LPS‐treated macrophages (Figure 6A), whereas the expression of proteins in the NF‐κB signalling pathway was not markedly changed (Figure 6B). In addition, co‐immunoprecipitation assay revealed that ER‐α36 indeed pulled down more p65 protein in the macrophages with ER‐α36 overexpression than that in cells infected with the empty lentivirus, as shown in Figure 6C. The results thus suggest that ER‐α36 regulates NF‐κB activity by influencing p65 subcellular localization.

We also performed a loss‐of‐function study using ER‐α36 siRNA to test the effects of ER‐α36 downregulation on calycosin activity in macrophages. The siR‐1 and the siR‐3‐transfected cells showed a significant reduction in ER‐α36 expression compared with the siR‐NC‐transfected cells, as shown in Figure 6D. ELISA assay revealed that ER‐α36 downregulation augmented IL‐6 production, which was attenuated by calycosin treatment (Figure 6E). Similarly, the expression of the NF‐κB signalling proteins was not affected in ER‐α36 knock‐down cells (Figure 6F). Meanwhile, ER‐α36 pulled down less p65 protein in the macrophages with knocked‐down level of ER‐α36 expression compared with the cells transfected with si‐NC, as revealed using co‐IP assay (Figure 6G). These findings indicate that the induction of ER‐α36 expression by calycosin contributes to calycosin inhibition of IL‐6 production in macrophages.

### 3.7 IL‐6 inhibitory activity of calycosin in TAMs contributed to its antitumour activity in vivo

**FIGURE 7:**
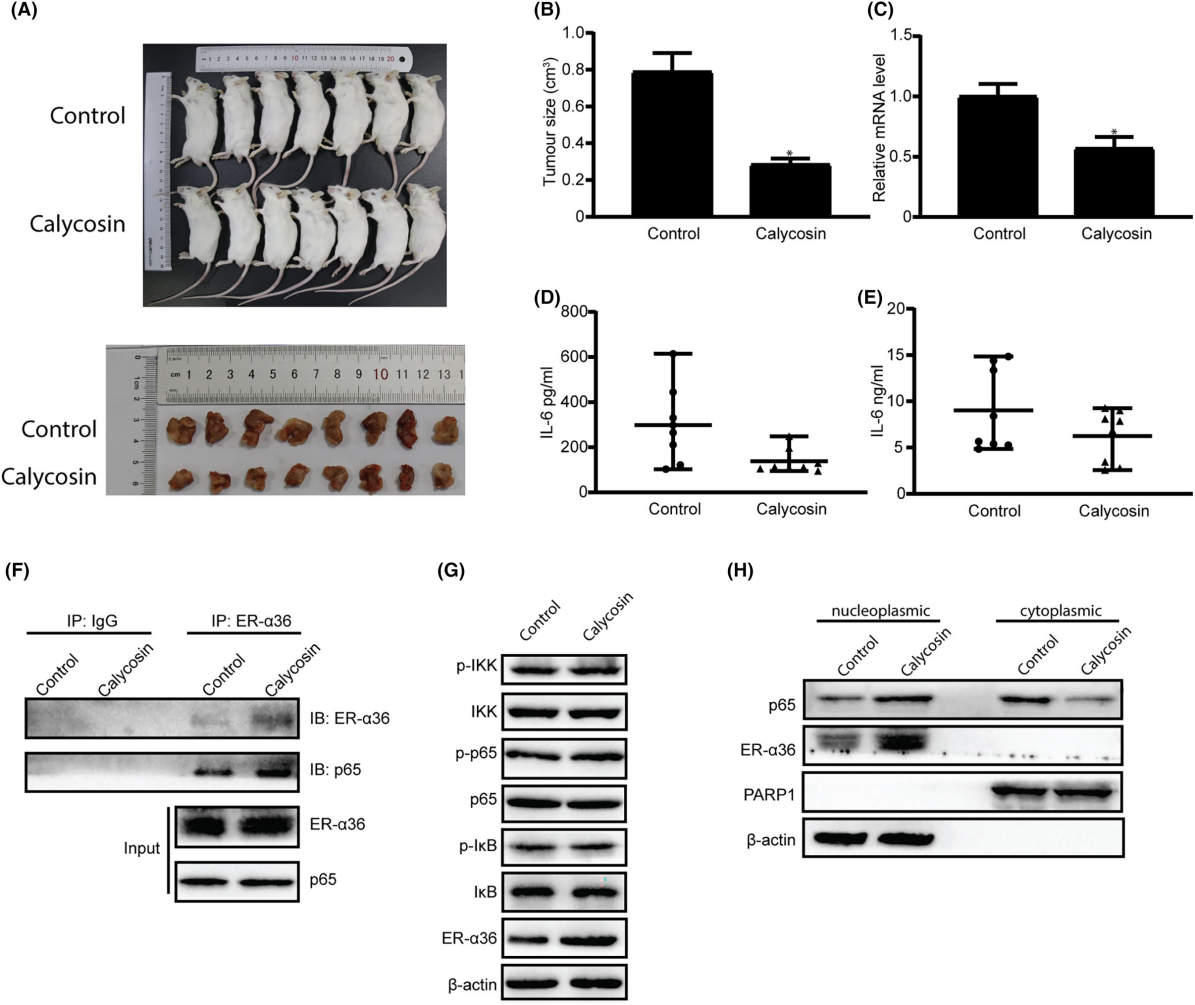
IL‐6 inhibition activity of calycosin in TAMs contributes to its antitumour activity in vivo. (A) The images of the mice carrying the tumours from mouse HCC Hepa1‐6 cells treated with or without calycosin (50 μg/kg), and the corresponding excised tumours. (B) The tumour sizes of the tumours derived from the Hepa1‐6 cells in the mice treated with or without calycosin. Values are expressed as means ± SEM. (*n* = 8, *p < 0.05 vs. the control group). (C) IL‐6 mRNA in the macrophages isolated from the tumour tissues was detected using RT‐PCR. The data are presented as means ± SEM (*n* = 8, *p < 0.05 vs. the control group). (D) ELISA of IL‐6 secreted into peripheral blood (*n* = 3 from three independent experiments). (E) ELISA of IL‐6 in tumour homogenates (*n* = 3 from three separate experiments). (F) Co‐immunoprecipitation experiments verified the interaction between ER‐α36 and p65. (G) Calycosin has no effect on the expression level of the NF‐κB signalling proteins in the macrophages isolated from the tumour tissues. (H) The nucleoplasmic and cytoplasmic separation assay of p65 proteins in the macrophages isolated from the tumour tissues of the mice treated with or without calycosin. PARP1 and β‐actin antibodies were used as nucleoplasmic and cytoplasmic markers, respectively.

To validate the IL‐6 inhibitory activity of calycosin, mouse Hepa1‐6 cells were inoculated into BALB/c mice to establish a subcutaneously implanted tumour model. Mice carrying xenograft tumours were treated with calycosin via intraperitoneal injection (50 μg/kg per day) for 30 days. The tumours from calycosin‐treated mice were substantially smaller than those in the control group treated with PBS (Figure 7A) and showed slower growth than those of the control group (Figure 7B), indicating that calycosin inhibits HCC tumour growth in vivo.

To confirm that the inhibition of IL‐6 production by TAMs is involved in the antitumour activity of calycosin, we isolated and purified macrophages from xenografted tumours and found that the IL‐6 mRNA level in these TAMs was downregulated in the calycosin group compared to those in the control group, as revealed in the RT‐PCR assay (Figure 7C). When the level of IL‐6 in peripheral blood and tumour homogenates was assessed using ELISA, we found that calycosin downregulated IL‐6 production (Figure 7D,E). Co‐immunoprecipitation assay also showed the interaction between p65 and ER‐α36 in the isolated macrophages (Figure 7F). Western blotting demonstrated that the expression level of NF‐κB signalling proteins did not change in the presence or absence of calycosin (Figure 7G). Calycosin treatment decreased the nuclear localization of p65 compared to that in mice treated with PBS, as shown in Figure 7H.

These results demonstrate that IL‐6 inhibitory activity of calycosin occurred in the TAMs of the mouse model, which contributed to its antitumour activity in vivo. Our results also indicate that ER‐α36 is involved in the calycosin activity.

## 4 DISCUSSION

At the cellular level, carcinogenesis is a multistep process involving the mutation and selection of tumour cells with progressively enhanced abilities to proliferate, survive, invade and metastasize.^28^ The TME often refers to the internal and external environments of tumour cells and comprises a mass of heterogeneous cell types, including immune cells, endothelial cells, fibroblasts and cancer cells. It is becoming increasingly clear that the development of this supporting niche is critical for tumour growth, diffusion and metastasis.^29^ Here, we first used the TIMER algorithm to examine the correlation between TAM infiltration and the survival of patients with liver hepatocarcinoma and found that patients with higher macrophage infiltration exhibited poorer survival than patients with lower macrophage infiltration. Moreover, a positive correlation between IL‐6 expression and tumour‐infiltrating macrophages was observed in these samples. We also demonstrated that IL‐6 secreted from macrophages stimulates the growth of HCC cells, which is consistent with a report that IL‐6 is associated with poor prognosis, recurrence and metastasis in various types of cancer.^30^ Thus, targeting IL‐6 production in TAMs may offer patients with cancer alternative and novel treatment options.

Isoflavonoids and isoflavones (also known as phytoestrogens) exhibit diverse biological activities, and among these, their anticancer effects are the most noteworthy. However, most studies on these agents have been performed directly in different cancer cells, proposing proliferation inhibition, cell cycle arrest, apoptosis induction and metastasis attenuation as the major underlying mechanism of their anticancer activities. Genistein, a phytoestrogen, has been shown to inhibit the stemness of breast,^31^ intestinal cancer^32^ and ovarian cancer^33^ by modifying the TME; however, little has been reported about the roles of other phytoestrogens in the TME.

Previously, oestrogens have been reported to reduce the production of proinflammatory cytokines in monocytes through ER‐α36.^20^ Calycosin also attenuated IL‐6 production.^34^ Here, we used molecular docking to predict calycosin binding affinity and energy to ER‐α36 and found the strongest binding of calycosin to ER‐α36 compared with formononetin and biochanin A. Our data thus strongly suggested that calycosin may inhibit IL‐6 production in macrophages through ER‐α36.

PBMCs from healthy donors and a monocyte THP‐1 cell line established from the peripheral blood of a patient with acute monocytic leukaemia^35^ were differentiated into macrophages using M‐CSF and PMA, respectively. The THP‐1 cell line has been widely used to study immune responses because the cells are not only in the monocyte state but also in the macrophage‐like state.^36^ As a specific LPS transmembrane receptor, TLR4 interacts with LPS to activate the LPS signal transduction pathways that enhance NF‐κB activity.^37^ Activated NF‐κB increases the transcription of several cytokines, such as TNF‐α and IL‐6.^38^ In this study, we found that calycosin had no effect on the expression level of NF‐κB signalling proteins but suppressed the NF‐κB transcriptional activity in a dual‐luciferase assay, suggesting that a mechanism other than direct regulation of NF‐κB protein expression is involved.

Previous studies have suggested an interaction of ER‐α36 with p65 in response to oestrogens.^20^ Here, we showed that calycosin upregulated ER‐α36 expression, promoted the interaction between p65 and ER‐α36 and augmented the retention of p65 in the cytoplasm, which ultimately inhibits NF‐κB transcription activity in macrophages treated with calycosin. These results suggest a molecular mechanism by which calycosin inhibits IL‐6 production by macrophages.

The involvement of ER‐α36 in the regulation of IL‐6 expression in macrophages was further confirmed in THP‐1 cells with forced expression of ER‐α36. Forced expression of ER‐α36 in THP‐1 cells significantly inhibited LPS‐induced IL‐6 production. More p65 subunits interacted with increased ER‐α36 expression and were presumably retained in the cytoplasm, which ultimately downregulated the NF‐κB transcription activity and IL‐6 expression. On the contrary, when ER‐α36 expression was downregulated using siRNA, it was found that IL‐6 secretion from macrophages was enhanced when ER‐36 expression was knocked down. Taken together, these results strongly indicated that ER‐α36, a novel variant of oestrogen receptor, is involved in the regulation of NF‐κB signalling pathway and then IL‐6 production.

Previous reports have shown that oestrogen represses tumour growth in human HCC cells.^39^ Here, we showed that calycosin suppressed tumour growth in BALB/c mice subcutaneously inoculated with mouse HCC Hepa1‐6 cells. We also showed that ER‐α36 interacted with p65 and reduced IL‐6 production in tumours in these TAMs. The result thus strongly suggested that calycosin inhibitory activity on IL‐6 expression involves ER‐α36, which is in good agreement with the in vitro results.

Notably, the calycosin concentration used in several cancer cell experiments reported previously is >5 μM that is often difficult to achieve in vivo. Thus, the reported effects of calycosin, such as growth inhibition, cell cycle arrest and apoptosis induction, may not be responsible for tumour growth inhibition observed in vivo. Here, the effect of calycosin was observed in the nanomolar range, suggesting that inhibition of the NF‐κB signalling pathway and cytokine production may be important in tumour growth inhibition.^12^

In conclusion, we demonstrated that calycosin inhibits LPS‐induced IL‐6 secretion from TAMs, indicating a role of ER‐α36 in the regulation of the NF‐κB signalling pathway. In addition to expanding our knowledge of ER‐α36, these findings could also help explain cancer development in which TAMs play a critical role. We suggest that the efficiency of the calycosin‐based strategy may be improved by combining it with anti‐IL‐6 therapy for cancer treatment.

## AUTHOR CONTRIBUTIONS

**Guoli Wu:** Methodology (equal); validation (equal); visualization (equal); writing – original draft (equal). **Guangying Qi:** Conceptualization (equal); project administration (equal); resources (equal). **Yu Liu:** Data curation (equal); formal analysis (equal); supervision (equal). **Jinfeng Gan:** Data curation (equal); formal analysis (equal); software (equal). **Chichu Xie:** Data curation (equal); methodology (equal); validation (equal). **Qi Wu:** Methodology (equal); visualization (equal). **Wei Cui:** Methodology (equal); validation (equal). **Chunhua Wang:** Writing – review and editing (equal). **Zhaoyi Wang:** Conceptualization (equal); funding acquisition (equal); project administration (equal); resources (equal); writing – review and editing (equal).

## FUNDING INFORMATION

Guangxi BaGui Scholars Program, No [2019]79.

## CONFLICT OF INTEREST STATEMENT

The authors declare no conflicts of interest.

## Supporting information


Figure S1.

Figure S2.

Figure S3.

Figure S4.

Appendix S1.

## Data Availability

A publicly available dataset was analysed in the current study and is available at http://www.ncbi.nlm.nih.gov/geo (GSE173120).
