# Supplementary figures and images for: ER‐α36 is involved in calycosin inhibition of IL‐6 production in macrophages
Source: J Cell Mol Med. 2023 Nov 16;28(1):e18037. doi: 10.1111/jcmm.18037 (PMC10805506; doi:10.1111/jcmm.18037)

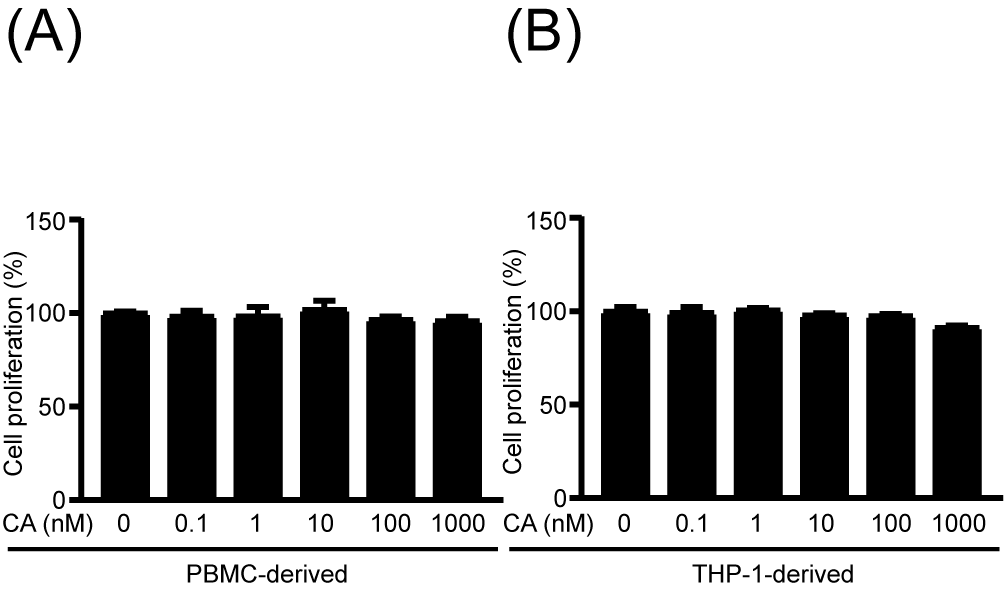

Supplement: Supplementary file 1 — Figure S1. [file JCMM-28-e18037-s001.tif]

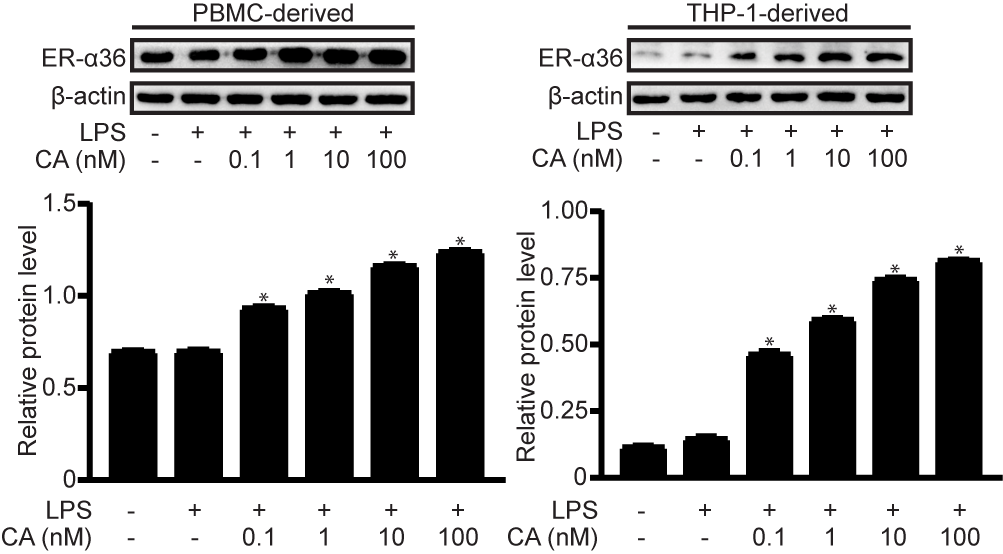

Supplement: Supplementary file 2 — Figure S2. [file JCMM-28-e18037-s005.tif]

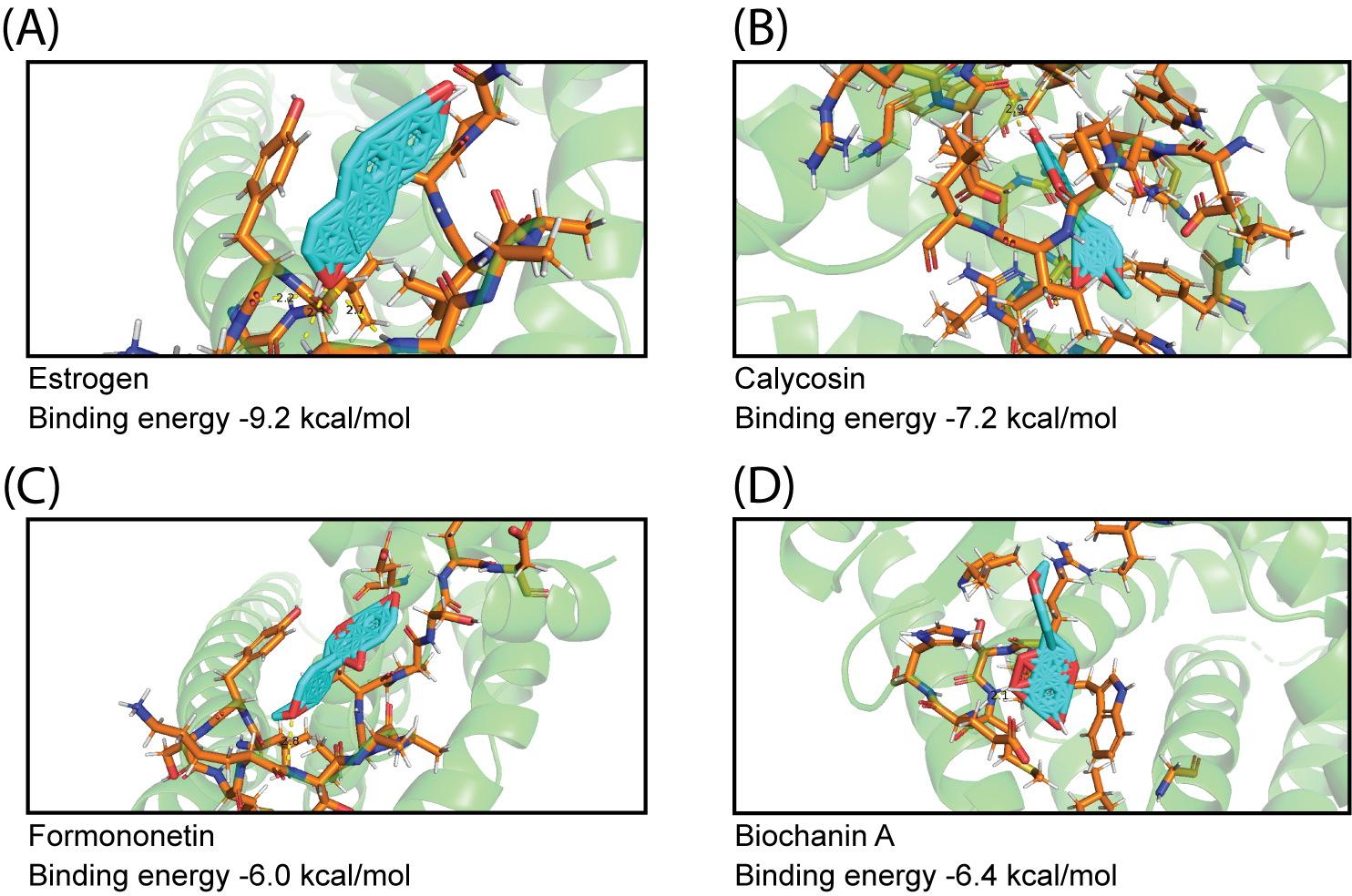

Supplement: Supplementary file 3 — Figure S3. [file JCMM-28-e18037-s002.tif]

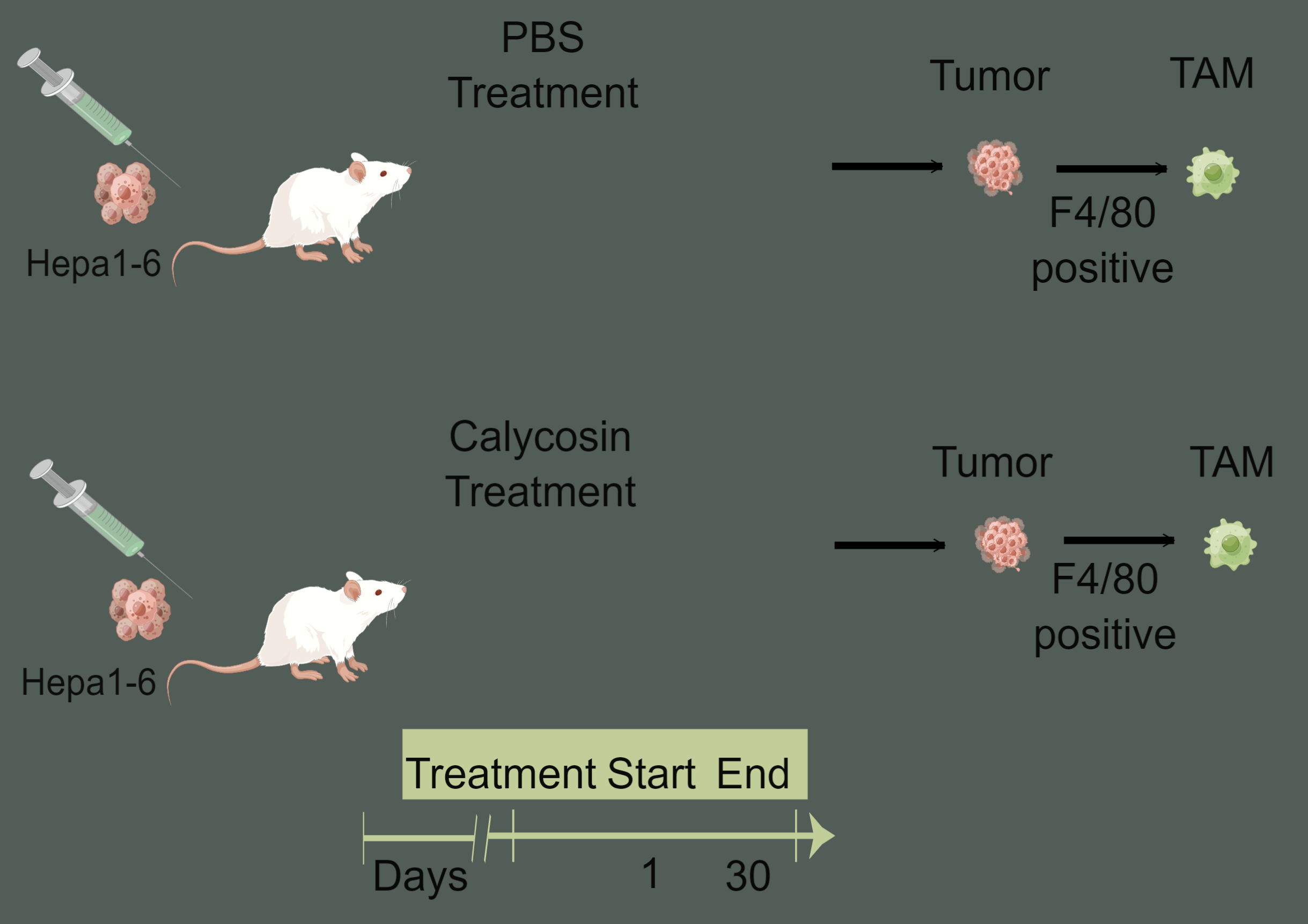

Supplement: Supplementary file 4 — Figure S4. [file JCMM-28-e18037-s003.tif]
