# Supplementary material for: ER‐α36 is involved in calycosin inhibition of IL‐6 production in macrophages
Source: J Cell Mol Med. 2023 Nov 16;28(1):e18037. doi: 10.1111/jcmm.18037 (PMC10805506; doi:10.1111/jcmm.18037)
Supplement: Supplementary file 5 — Appendix S1. [file JCMM-28-e18037-s004.docx]

***Supplementary material***

**1. Supplemental table：the primers used for RT-PCR**

| Genes | Forward primer (5’-3’) | Reverse primer (5’-3’) |
| --- | --- | --- |
| *ACTB* | CATGTACGTTGCTATCCAGGC | CTCCTTAATGTCACGCACGAT |
| *IL6* | CCTGAACCTTCCAAAGATGGC | TTCACCAGGCAAGTCTCCTCA |
| *Actb* (*Mm*) | GGCTATGCTCTCCCTCACG | GAGCAACATAGCACAGCTTCTCTTT |
| *Il6* (*Mm*) | CTGATGCTGGTGACAACCAC | GGTCTGTTGGGAGTGGTATCC |

**2. siRNA sequence**

The siRNA sequences for si-ER-α36 and si-NC were as follows:

si-ER-α36 1 (siR-1)

UUAACCGUACCACUCUGCUGA
UCAGCAGAGUGGUACGGUUAA
si-ER-α36 2 (siR-2)

UACUUGUAGCAAAUAAGCACG
CGUGCUUAUUUGCUACAAGUA
si-ER-α36 3 (siR-3)

UUGAUGCCAAUAGGUACUGAA
UUCAGUACCUAUUGGCAUCAA
si-ER-α36 4 (siR-4)

UUGAUUCUGGAAACUUGUCUC
GAGACAAGUUUCCAGAAUCAA

siRNA control (siR-NC)

UUCUCCGAACGUGUCACGUUU
ACGUGACACGUUCGGAGAAUU

**Supplemental Figures**

**Figure S1.** Cells treated with calycosin was examined using the MTT assay (A&B). Data are shown as the mean ± SEM of three independent experiments, ^*^ p < 0.05 vs. the control group (LPS- and calycosin-), ^#^ p < 0.05 vs. the LPS group (LPS 1 μg/mL and calycosin-).

**Figure S2.** Western blotting of ER-α36 expression in PBMC- and THP-1-derived macrophages treated with calycosin in the presence of LPS. Protein bands were semi‑quantified with densitometric analysis. Data are shown as the mean ± SEM of three independent experiments, ^*^ p < 0.05 vs. the control group (LPS-, and calycosin-).

**Figure S3.** Partial diagram of molecular docking: (**A**). ER-α36-oestrogen, (**B**). ER-α36-Calycosin, (**C**). ER-α36-formononetin, (**D**). ER-α36-biochanin A. As well as their binding energies.

**Figure S4.** Schematic showing the flow of animal experiment.

**Supplemental Method and Materials**

**Western blotting**

The different groups of cells were lyzed in RIPA lysis buffer (Solarbio) and after centrifuged, the concentrations of total proteins in individual cell lysate samples were quantified using a BCA kit (Beyotime). The cell lysates (30 µg/lane) were separated by SDS-PAGE on 12% gels and transferred to PVDF membranes (Merck-Millipore, Darmstadt, Germany). After being blocked with 5 % fat-free dry milk in TBST, the membranes were incubated with the primary antibodies overnight at 4 ℃ and the primary antibodies included those against β‑actin, IKK, p65, IκB, p-IKK, p‑p65, p-IκB and ER-α36. The membranes were washed and the bound antibodies were detected with HRP‑conjugated secondary anti‑rabbit antibodies or anti‑mouse antibodies. The immunocomplex was visualized using the chemiluminescence ECL kit (Life-iLab, Shanghai, China). The band intensities were semi‑quantified by densitometric analysis using the Image J program.

**Cell staining and immunofluorescence**

The different groups of cells were fixed in 4% paraformaldehyde (Solarbio) and permeabilized with 0.5% of Triton X-100 (Solarbio), followed by blocking with 5% BSA (Solarbio) for 1 h. The cells were stained with primary antibodies against p65 and ER-α36 overnight, and stained with [CoraLite594](https://www.ptgcn.com/products/CoraLite594-conjugated-Donkey-Anti-Mouse-IgG-H-L.htm)-anti-mouse (red) and FITC-anti-rabbit (green) secondary antibodies (Proteintech) for 1 h. The immunofluorescent signals were examined under a confocal microscope (Leica Microsystems, Germany) at 63×oil objective.

**Cell viability assay**

PLC/PRF/5, BEL-7402 cells (5×10^3^ cells/well) were cultured in 96‑well plates. PLC/PRF/5 and BEL-7402 were treated in triplicate with the calycosin-treated conditional medium for 48 h. During the last 4-h culture, individual wells of cells were added with 20 µl of MTT solution (Solarbio). The absorbance of individual wells at 490 nm was measured using a microplate reader (BioTek, VT, USA). The same number of induced macrophages were treated with different concentrations of calycosin for 48 h. The impact of calycosin on the viability of induced macrophages was determined by MTT assays. Cell proliferation rate was calculated according to the formula, (OD of experimental group / OD of control group) × 100%.

**ELISA**

The level of IL-6 in individual samples was quantified by ELISA using a specific kit (DAKEWE, Beijing, China). The experimental and control samples were tested in triplicate simultaneously. The concentrations (pg/ml) of IL-6 were calculated by a microplate reader (BioTek, VT, USA) at a 450 nm maximum absorption wavelength, according to the standard curve established using recombinant IL-6 provided.

**ChIP (chromatin immunoprecipitation)**

The different groups of cells were harvested, washed twice with PBS, and fixed in 1% formaldehyde (Solarbio) for 15 min, followed by treatment with 0.125 M glycine (Solarbio) to terminate the cross-linking reaction. The cells were lyzed in 2 ml of lysis buffer (Santa Cruz Biotechnology) and their nuclei were extracted and centrifuged at 1,500 *g* for 10 min. The nuclear pellets were resuspended with 1.5 ml of high salt buffer (Santa Cruz Biotechnology), and centrifuged at 12,000 *g* for 20 min. Their supernatants (chromatin solutions) were sonicated and reacted with anti-Rpb1, anti-p65, or control IgG overnight. The immune-complexes were precipitated with protein A/G agarose beads (Santa Cruz Biotechnology), washed and incubated in a 67 ℃ water bath, followed by finally centrifuging at 12,000 *g*. The DNA in the nuclear extracts were extracted using 600 µl of DNA extraction solution (Solarbio) and precipitated using 1.2 ml of absolute ethanol and 1 µl of Glycogen (Beyotime) at -20 ℃ for 2 h. The precipitated DNA was washed with 70 % ethanol and dissolved in H_2_O for ChIP analysis.

The primers for the IL-6 promoter: forward 5′-AAATGCCCAACGGAGGTCAC-3′; reverse 5′-TTCCCTCAGGATGGTGTCTC-3′.

**Immune cell infiltration analysis**

The macrophage infiltration in hepatocarcinoma and its relationship with the patient survival were estimated by TIMER2.0 (http://timer.comp-genomics.org/) as well as the correlation between macrophage infiltration and IL-6 expression.

**Molecular docking**

The 3D protein conformations of 6VPF (3D structure of ER-α) were acquired from the PDB database. and edited using NotePad++ software. The SDF format files of estrogen, calycosin, formononetin, and biochanin A were obtained from the PubChem platform. The potential interactions among them were analysed using the AutoDock Tool software and their binding energies were acquired using autogrid4 and autogrid4 software, followed by visualizing using the PyMol software.

**Acquisition of differentially expressed genes**

The [GSE173120](https://www.ncbi.nlm.nih.gov/geo/query/acc.cgi?acc=GSE173120) dataset was obtained from the GEO database, and it includes transcriptomes from three human cervical cancer caski cell samples as the negative control (NC) and three si-ER-α36-transfeccted caski cell samples. The differentially expressed genes (DEGs) between two groups of cells were identified using the NetworkAnalyst. Based on a *P* value of 0.05 and |log_2_FC| > 0.5, The DEGs were analysed by volcano plot and heatmap using bioinformatics (bioinformatics.com.cn). The potential functions and the relevant signaling pathways of DEGs were analysed by GO and KEGG enrichment.
